# Transcriptomic Characterization of Male Formosan Pangolin (*Manis pentadactyla pentadactyla*) Reproductive Tract and Evaluation of Domestic Cat (*Felis catus*) as a Potential Model Species

**DOI:** 10.3390/ani14172592

**Published:** 2024-09-06

**Authors:** Laura Orama Méar, IShin Tseng, Kuei-Shien Lin, Chia-Lin Hsu, Szu-Hua Chen, Pei-Shiue Tsai

**Affiliations:** 1 Graduate Institute of Veterinary Medicine, National Taiwan University, Taipei 10617, Taiwan; r10629024@ntu.edu.tw; 2 Department of Reproduction Biology, Leibniz Institute for Zoo & Wildlife Research, Alfred-Kowalke Rd., No. 17, 10315 Berlin, Germany; 3 Institute of Epidemiology and Preventive Medicine, National Taiwan University, Taipei 10002, Taiwan; d12849007@ntu.edu.tw; 4 Taiwan Biodiversity Research Institute, Nantou 552005, Taiwan; sds0308@tbri.gov.tw (K.-S.L.); szuhua@tbri.gov.tw (S.-H.C.); 5 Department of Veterinary Medicine, National Taiwan University, Taipei 10617, Taiwan; b09609005@ntu.edu.tw; 6 Research Center for Developmental Biology and Regenerative Medicine, National Taiwan University, Taipei 10617, Taiwan

**Keywords:** pangolin, male reproductive tract, gene database, cat, conservation, endangered species

## Abstract

**Simple Summary:**

The Formosan pangolin (*Manis pentadactyla pentadactyla*) is a rare and enigmatic species endemic to Taiwan. Due to its declining population and elusive behavior, little is known about its reproduction. This study created a genomic database of the male pangolin’s reproductive tract, identifying key genes and pathways in the testis and epididymis. Comparing testicular genes expression between pangolins and domestic cats, we suggested that the cat is not a suitable model for studying pangolin reproduction due to significant differences. These insights advance our understanding of male pangolin reproduction and may help in the development of future artificial reproductive techniques for pangolins.

**Abstract:**

The Formosan pangolin (*Manis pentadactyla pentadactyla*) is an endemic animal of Taiwan. Due to their reduced population and behavior, very little is known about this enigmatic species. To unravel male pangolin reproduction, in the present study, we built a complete genomic database of the male Formosan pangolin reproductive tract and revealed highly expressing genes as well as critical signaling pathways and their associated biological processes in both the testis and the epididymis. Moreover, we evaluated the domestic cat (*Felis catus*) as a potential model species for male pangolin reproduction by comparing their testicular transcriptomes. We demonstrated a clear tissue-specific gene expression supporting the unique biological signature of each reproductive tissue and identified critical genes of the different reproductive organs. Pathway enrichment analysis revealed unique pathways in the testis as well as a clear epididymal transition. Furthermore, domestic cats, despite being the closest domestic species to pangolin, demonstrated their unfitness as a male reproduction model species as clear differences in spermatid differentiation and metabolism were observed. These results enable a better understanding of male pangolin reproduction characteristics and may inspire improvements in in Formosan pangolin conservation strategies.

## 1. Introduction

Pangolins belong to the Pholidota order and are recognized for their armored exterior composed of keratin scales [1]. They are termite- or ant-eating nocturnal mammals and are mostly habit in the mountains, forests, or shrubbery surroundings in tropical or subtropical countries. Despite their ecological importance, pangolins face severe threats that have propelled them into the critically endangered IUCN category [2]. Habitat loss, driven by human activities, such as deforestation, urbanization, and agricultural expansion, encroach upon the pangolin’s natural habitats, diminishing available resources and triggering population decline [2,3]. However, the most ominous threat arises from roadkill, and illegal hunting and trading, where the pangolin’s scales are believed to possess medicinal properties in traditional Asian medicine [4]. As conservationists strive to unravel the complexities surrounding these remarkable creatures, urgent efforts are required to protect their habitats, combat illegal wildlife trade, and raise awareness about the importance of preserving pangolins.

Despite nine species of pangolin identified, including the Chinese pangolin (*Manis pentadactyla*), knowledge regarding their reproductive biology is not well understood. The Formosan pangolin, endemic to Taiwan and a subspecies of the Chinese pangolin, is an elusive and endangered species indigenous to the island and is the only pangolin subspecies found in Taiwan [5]. The Formosan pangolin occupies a crucial ecological niche within Taiwan’s diverse ecosystems; however, their nocturnal lifestyle and underground activities during the daylight have added to the difficulties in researching them [4]. Earlier reports stated some successes in breeding Sunda (*Manis javanica*) pangolins [6]; however, the breeding of Formosan and/or Chinese pangolins in zoo captive populations have been less successful [7,8]. Optimized breeding protocol in captive pangolin populations relies on appropriate nutritional formulation, veterinary care, and, most importantly, a comprehensive understanding of their reproductive characteristics [7,9]. Earlier studies indicated that, similar to many farm animals, serum progesterone could be used as an indication of pregnancy success in pangolins, since serum progesterone in pregnant pangolins remained at 10–55 ng/mL with a peak at 47.6 ng/mL, whereas basal values between 1.99 ± 1.62 ng/mL and 2.27 ± 1.64 ng/mL were detected in non-pregnant pangolins [10]. Other observational studies showed that the gestation length of zoo-captive Formosan pangolins was 318 to 372 days [10,11]; however, whether the abovementioned information is valid for wild populations remains inconclusive. Apart from the reported reproductive characterizations in females, only an isolated paper regarding male pangolin reproduction has been published [12]. In that study, published by our group, we characterized, for the first time, the distinct anatomical structure of the Formosan pangolin reproductive tract and the unique morphology of pangolin sperm cells; moreover, we also demonstrated the presence and distribution of epididymal proteins, V-ATPase subunit 2, which have been suggested to be critical for maintaining the ion homeostasis of the male reproductive tract in other mammals. Due to limited knowledge about pangolin reproduction, the use of model species will be necessary to develop artificial reproduction techniques suitable for pangolin. Order Carnivora is the closest order to pangolins; domestic carnivores (*Felis catus* and *Canis lupus familiaris*) are, therefore, the most appropriate model species [13]. However, whether the taxonomical differences influence the fitness of these model species in reproduction is yet to be determined. To shed light on the similarities and differences between an animal model and species of interest, and to validate the suitability of a particular animal model to be used for studying species of interest, transcriptome analysis can be used to give a highly precise description at the genomic level [14]. For example, transcriptome profiling has previously been used to assess the adequacy of the mouse as an animal model for humans [15] and evaluate cryptorchidism animal models [16].

To further unravel male pangolin reproduction, in the present study, we build up a complete genomic database of the male Formosan pangolin reproductive tract and reveal highly expressing genes as well as critical signaling pathways and their associated biological processes in both the testis and epididymis. Moreover, we evaluate the fitness of the domestic cat (*Felis catus*) as a model species for male pangolin reproduction by comparing their testicular transcriptomes. This information enables a better understanding of male pangolin reproduction characteristics and may inspire cross-species modeling for the improvement of conservation strategy in Formosan pangolins (*Manis pentadactyla pentadactyla*).

## 2. Materials and Methods

### 2.1. Chemicals and Reagents

All chemicals were obtained from Sigma-Aldrich (St. Louis, MO, USA) unless stated otherwise.

### 2.2. Reproductive Tissue Collection

Reproductive tissues of six adult male Formosan Pangolin (*Manis pentadactyla pentadactyla*) with body weight > 2350 g, body length > 50 ± 5 cm, and tail length > 33 ± 2 cm were collected from road-killed individuals that were sent to the Taiwan Biodiversity Research Institute during the pangolin breeding period (November to March) between 2021 and 2023 (Supplementary Table S1). Within 3 h of the reported found/death time, a necropsy was performed by a certified veterinarian, and male reproductive organs were collected for further analysis. Both the testes and the epididymis were dissected from the animal upon necropsy procedures; tissues were either flash-frozen with liquid nitrogen and stored at −80 °C before RNA extraction or fixed with 10% neutralized formalin overnight for routine paraffin-embedding procedure. Reproductive tissues from three adult male domestic cats (*Felis catus*) were obtained through routine castration with owners’ consensus for the use of research purposes (Supplementary Table S2). After castration surgery, the reproductive tissues were kept at 4 °C to be transported to the laboratory. Testes and epididymis were dissected rapidly, and tissues were flash-frozen with liquid nitrogen before RNA extraction.

The acquisition and the use of tissue materials was followed by the regulation and approval of the animal welfare committee of Taiwan Biodiversity Research Institute (IACUC#112022) and was monitored under the guidance of the animal welfare committee of Taiwan Biodiversity Research Institute, Taiwan.

### 2.3. RNA-Seq Library Construction and Bioinformatic Analyses

Tissues from the male pangolin reproductive tract, including the testis (*n* = 6, pangolin testis, PT) and epididymis (*n* = 5, pangolin epididymis, PGE), were subjected to next-generation sequencing (RNAseq) and bioinformatic analyses. As the epididymis is known to exhibit regional-specific biological function and gene expression profile, distal (caput/corpus, PGE-1), and proximal (cauda, PGE-2) epididymis were manually separated for independent analysis. Total RNA was subjected to cDNA synthesis and NGS library construction using the Universal Plus mRNA-Seq Library Preparation Kit (Tecan Genomics, San Carlos, CA, USA). As no current database was available for library construction or comparative analysis for pangolin species, we constructed a first-ever pangolin male reproductive tract genomic library by collecting all ionized sequences and reads from the subjected samples and forming the basis of the pangolin reproductive tract genome library. By comparing the raw reads of each individual sample with the abovementioned self-constructed genome library, further analysis and comparisons were conducted.

The quality and average length of the sequence library for each sample were assessed using a Bioanalyzer (Agilent Technologies, Santa Clara, CA, USA) and the DNA 1000 kit, respectively. The indexed samples were pooled equimolarly and sequenced on Illumina NovaSeq 6000 (150 bases, paired-end reads) (Illumina, San Diego, CA, USA). Raw reads were quantified using CLC Genomics Workbench v.10 software (QIAGEN, Venlo, The Netherlands). Adaptor sequences and bases with low quality or ambiguities were trimmed. The trimmed reads were used to perform de novo assembly using Trinity (v2.9.1) [17]. The contigs were using CD-HIT (Version: v4.6) to cluster [18]. The clustered contigs were blasted against the NCBI database using BLASTN and DIAMOND [19]. Also, the contigs were annotated for Gene Ontology (GO) using FastAnnotation [20], and pathway using KEGG Automatic Annotation Server (KAAS) [21]. The trimmed reads were mapped to the clustered contigs using CLC Genomics Workbench. The mapping parameters were the following: mismatch cost 2, insertion cost 3, deletion cost 3, length fraction of 0.5, and similarity fraction of 0.8. The expression values were calculated as FPKM (fragments per kilobases per million). The differential gene expression between two or more conditions is based on the fold change of FPKM value. The genes with 2-fold change were further analyzed. KEGG database [21,22] was used in pathway enrichment analysis, and the pathway map was plotted by the pathview [19] package in R. Gene Ontology (GO) enrichment was analyzed by GO-TermFinder.

### 2.4. Data Sorting/Filtering

**Figure 1:**
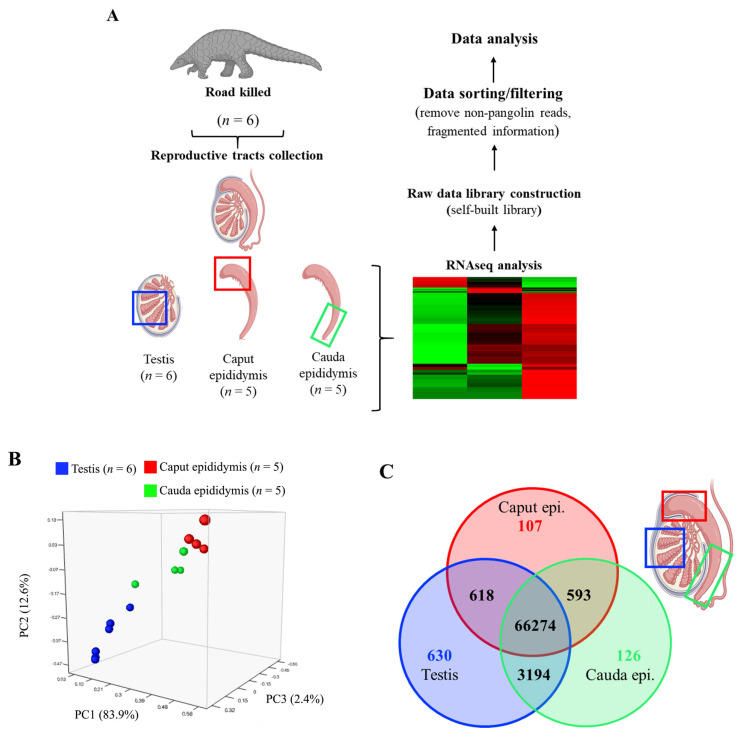
General overview of the testis, caput, and cauda transcriptome. (**A**) Workflow for the reproductive tract tissue collection and the creation of the genomic database of the pangolin male reproductive tract; (**B**) principal component analysis of pangolin male reproductive tract showed clear separation on the general gene expression profiles of the testis and epididymis; (**C**) Venn diagram for cross-tissue comparison.

As shown in Figure 1A, 6 individual testicular samples and 5 individual caput and 5 cauda epididymal regions (in total, 16 samples) were subjected to RNAseq analysis. Upon acquisition of the database, we obtained a total number of 279,836 transcripts; however, many gene annotations were not from the pangolin (Manidae family) or were only detected in a few individuals. As this is, to our knowledge, the first genomic database of the male pangolin reproductive tract ever presented, we applied the most stringent criteria for the subsequent analysis. We first filtered out transcripts that were not associated with the Manidae family using the existing NCBI nucleotide database (October 2023) to ensure the transcripts were from the default species (pangolin), and this gave us a total of 167,405 transcripts. Secondly, a *p*-value greater than 0.05 or missing data in any of the following columns in the “subject id” “Annotation”, and “FPKM” were excluded for the subsequent analysis. We also observed that some transcripts were only detected in one or two individuals. To avoid over-interpretation of the results, we further removed the transcripts that were not detected in all individuals; in other words, when transcripts were detected in all subjected samples (6 from the testis, 5 from the caput epididymal, and 5 from the cauda epididymal), the transcripts were processed further for the subsequent analysis (Figure 1). This additional filtering of the dataset resulted in a total number of 71,542 transcripts. Common and tissue/regional-specific genes and their paired comparisons were further carried out using R software 4.4.0. For statistical analysis, we utilized version 4.0.3 of the software with the “readxl”, “dplyr”, and “VennDiagram” packages, setting the statistical significance at a *p*-value threshold of less than 0.05.

### 2.5. Inter-Species RNAseq Analysis

Tissues from the reproductive tract of the male domestic cats, including the testis (n = 3), caput epididymis (n = 3), and cauda epididymis (n = 3), were collected for RNAseq and inter-species bioinformatic analyses. RNA extraction was performed using RNeasy Mini Kit (QIAGEN, Venlo, The Netherlands). Total RNA was subjected to cDNA synthesis and NGS library construction using the Universal Plus mRNA-Seq Library Preparation Kit (Tecan Genomics, San Carlos, CA, USA).

CLC Genomics Workbench v10 was used for sequence trimming (150 base pair as one unit) to avoid low-quality sequences influencing data analysis to ensure and validate the raw data obtained. To identify overlapping genes between the two species, “gene blasx” from the pangolin datasets was used to identify the genes with the same function in the house cat dataset. The house cat analysis did not define the isoform type of each gene. Therefore, the pangolin transcript, previously obtained with isoform-specific definition, was modified to exclude isoform precision, significantly reducing its size, to allow for fair comparison. Once all the genes with comparable/same functions were identified in both datasets, normalization was further performed. Normalization of gene expression was carried out with fragments per kilobase per million (FPKM). Individual gene expression level was normalized with its own FPKM of the *ACTB* (housekeeping gene, beta-actin) before a cross-species normalization was conducted. To compare the differences in gene expression, the reference sequence of cat (*Felis catus*; Fca126_mat1.0) was used as the basis to calculate the FPKM values of each identified gene. Genes of unknown function were excluded from the comparison to focus on the functional annotations. After gene expressions were normalized between pangolin and house cats, RNAseq analyses, such as differentially expressed genes (DEGs), volcano plots, and pathway analysis, were subsequently made. Further functional comparative analyses were performed using DAVID analysis to obtain the main general biological processes of each gene group (non-differentially expressed, upregulated, downregulated, and pangolin unique). The gene list of the biological processes that possessed the highest number and gene enrichment *p*-value were then separated for further analysis. The subsequent analysis consisted of STRING protein analysis, which allows for the study of precise biological processes, tissue expression, and subcellular location.

## 3. Results

### 3.1. RNAseq Analysis Reveals Tissue-Specific Gene Expressions of Pangolin Male Reproductive Tract

Principal component analysis (PCA) showed a distinct gene expression pattern between the testis, caput, and cauda epididymis with relatively small variations between different individuals as apparent separated clusters of different tissues were observed (Figure 1B); surprisingly, PCA analysis showed a more similar gene expression profiles between the testis (labeled in blue) and cauda epididymis (labeled in green) when compared with the differences between the testis and caput epididymis (labeled in red) (Figure 1B). This finding was further confirmed when cross-tissue comparisons were performed. As shown in the Venn diagram, testis and cauda epididymis shared 3194 common genes, while testis shared only 618 common genes with the caput epididymis (81% less than cauda) (Figure 1C). Besides 66,274 genes that are commonly expressed in all three tissues (testis, caput, and cauda epididymis), we also identified 630 testis-specific (blue circle), 107 caput-specific (red circle), and 126 cauda-specific (green circle) expressing genes (Figure 1C).

### 3.2. The Top 5 Highly Expressed Genes Identified in Each Region Support the Unique Biological Signatures of Each Reproductive Tract Section

**Figure 2:**
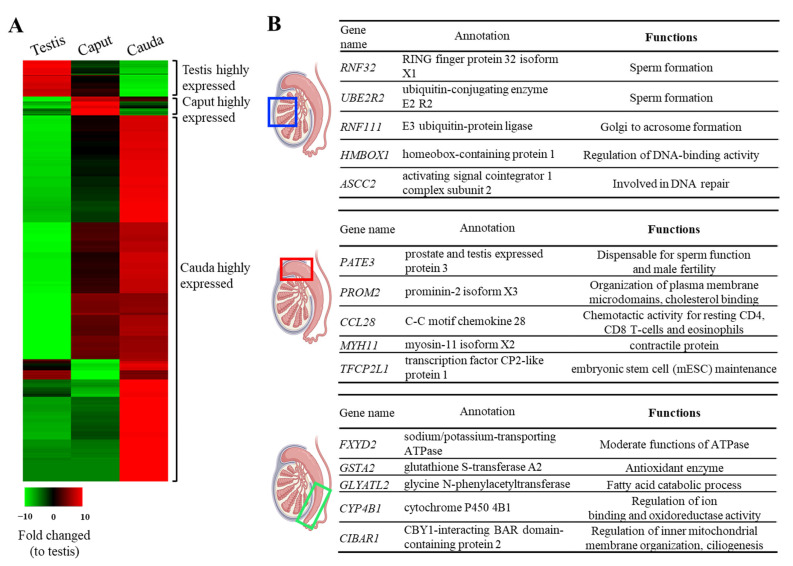
Tissue-specific gene expression profiles. (**A**) Heat map analysis of highly expressed genes across the male reproductive tract; using testicular genes as the basis of comparison, tissue- and regional-specific highly expressed genes were revealed; within commonly expressed genes, a large proportion of the genes were highly expressed in the cauda and only a small fraction of genes were highly expressed in the testis or caput epididymis. (**B**) The list of the top 5 highest-expressing genes in different reproduction organs. Blue box: testis; Red box: Caput epididymis; Green box: Cauda epididymis.

RNAseq analysis showed that among the 66,274 commonly expressed genes, a large portion of genes was highly expressed in the cauda epididymis when compared with testis, and only a small fraction of genes was highly expressed in either testis or caput epididymis (Figure 2A). Heatmap analysis indicated a pattern of regional-dependent highly expressing genes; therefore, we further explored the functions of these genes. Not surprisingly, the top testicular expressing genes were mostly associated with spermatogenesis, such as RING finger protein 32 (*RNF32*), ubiquitin-conjugating enzyme E2 R2 (*UBE2R2*), E3 ubiquitin–protein ligase (*RNF111*), homeobox-containing protein 1 (*HMBOX1*), and activating signal co-integrator 1 complex subunit (*ASCC2*) (Figure 2B, upper panel). Interestingly, in caput epididymis, the top tier expressing gene, *PATE3* (prostate and testis expressed protein 3) has been shown to be dispensable for sperm function and male fecundity as *PATE3* knockout mice showed normal fertility (Figure 2B, middle panel) [23]. Nevertheless, other genes identified in either the caput epididymis (i.e., *PROM2*, *CCL28*, *MYH11*, *TFCP2L1*) or cauda epididymis (i.e., *FXYD2*, *GSTA2*, *GLYATL2*, *CYP4B1*, *CIBAR1*) and their associated proteins are likely to contribute to the maintenance of the epididymal microenvironment or are responsible for plasma membrane modification and sperm movement (Figure 2B, middle and lower panels).

### 3.3. Pathway Enrichment Analysis Reveals Unique Pathways in the Testis but Common Signaling Pathways throughout the Epididymis

**Figure 3:**
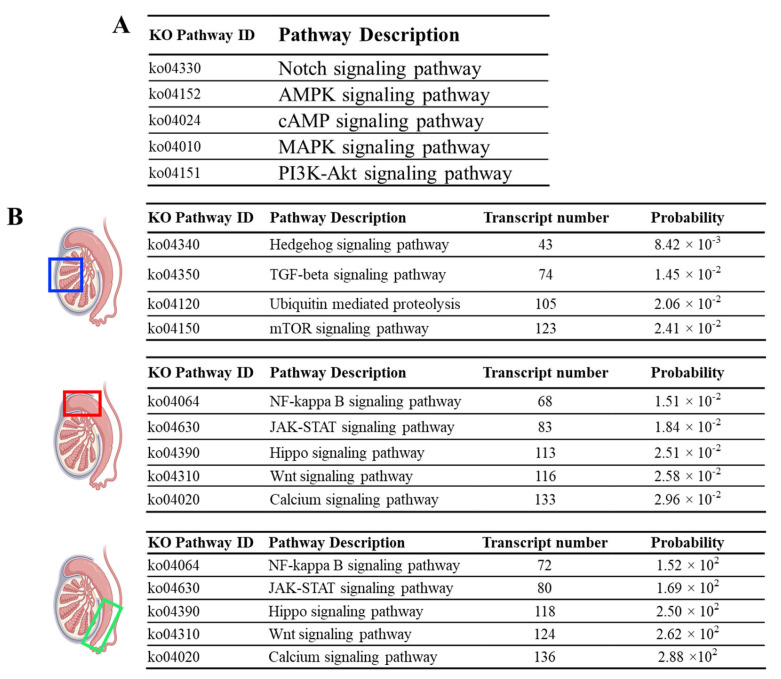
Pathway analysis of the RNAseq result. (**A**) Top active signaling pathways that were commonly detected in the testis, caput, and cauda epididymis. (**B**) Tissue-specific signaling pathways that are highly active in different reproductive organs. While testis showed some unique active signaling pathways, no apparent differences in terms of active signaling pathways can be detected between caput and cauda epididymis. Blue box: testis; Red box: Caput epididymis; Green box: Cauda epididymis.

Upon pathway analysis, we detected several signaling pathways—such as the Notch, 5′ adenosine monophosphate-activated protein kinase (AMPK), cyclic adenosine monophosphate (cAMP), mitogen-activated protein kinase (MAPK), and Phosphoinositide 3-kinases-Akt (PI3K-Akt) signaling pathways—that were commonly active throughout the pangolin male reproductive tract (all significantly active in the testis, caput, and cauda epididymis) (Figure 3A). Interestingly, unlike other domestic or rodent species, we observed no apparent differences in terms of highly active signaling pathways between caput and cauda epididymis, as the top-ranked signaling pathways in both caput and cauda epididymis were NF-kappa B signaling pathway, JAK-STAT signaling pathway, Hippo signaling pathway, Wnt signaling pathway, and calcium signaling pathway (Figure 3B). Nevertheless, testis exhibited some unique pathways, such as the Hedgehog, TGF-beta, Ubiquitin-mediated proteolysis, and mTOR pathway (Figure 3B).

### 3.4. Specific Biological Activities Are Significantly Upregulated upon Epididymal Transition

**Figure 4:**
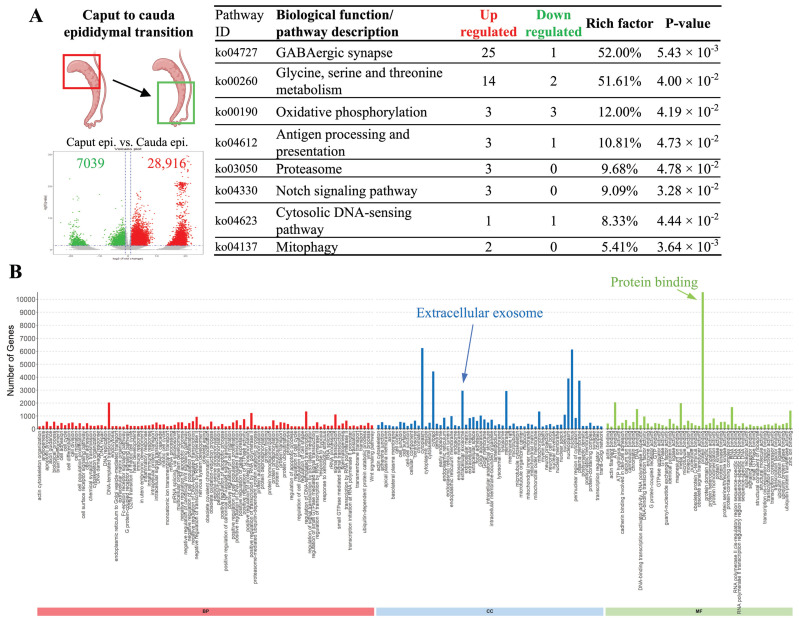
Comparisons of differentially expressed genes between caput and cauda epididymis. (**A**) When commonly expressed genes from caput and cauda epididymis were compared, we observed 28,916 genes that were upregulated and only 7039 commonly expressed genes were downregulated. (**B**) Gene Ontology analysis of upregulated genes in the caput epididymis showed a significant number of genes identified were originated from extracellular vesicles/exosomes. Molecular function analysis showed that the highly upregulated genes in the caput epididymis were mostly known to mediate protein binding processes.

Interestingly, despite the fact that in pathways analysis (Figure 3B), we observed no differences in the top 10 highly upregulated signaling pathways between caput and cauda epididymis, genes responsible for amino acid metabolism, oxidative phosphorylation, antigen processing and presentation, proteasome, mitophagy activities, and the Notch and cytosolic DNA-sensing pathway were mostly upregulated in the caput epididymis, suggesting more active cellular activities were taking places at the proximal (caput) region of the epididymis rather than the distal (cauda) epididymis (Figure 4A). Moreover, when individual gene expression was examined, we observed 28,916 genes that were upregulated and only 7039 common genes were downregulated when compared caput with cauda epididymis (Figure 4A). Gene Ontology (GO) analysis showed that besides the expected cellular component of cytoplasm, nucleoplasm, and plasma membrane, a significant number of genes identified (*p* = 0.04, 781 annotations) were known to present within the extracellular vesicles/exosome (Figure 4B, indicated by blue arrow). Molecular function-wise, the highly upregulated genes were mostly known to mediate protein binding processes (*p* = 9.28 × 10^−7^ with 2590 annotations) (Figure 4B).

### 3.5. Cross-Species Comparisons Demonstrate 50% Transcriptome Variations between Pangolin and Domestic Cat Testes

**Figure 5:**
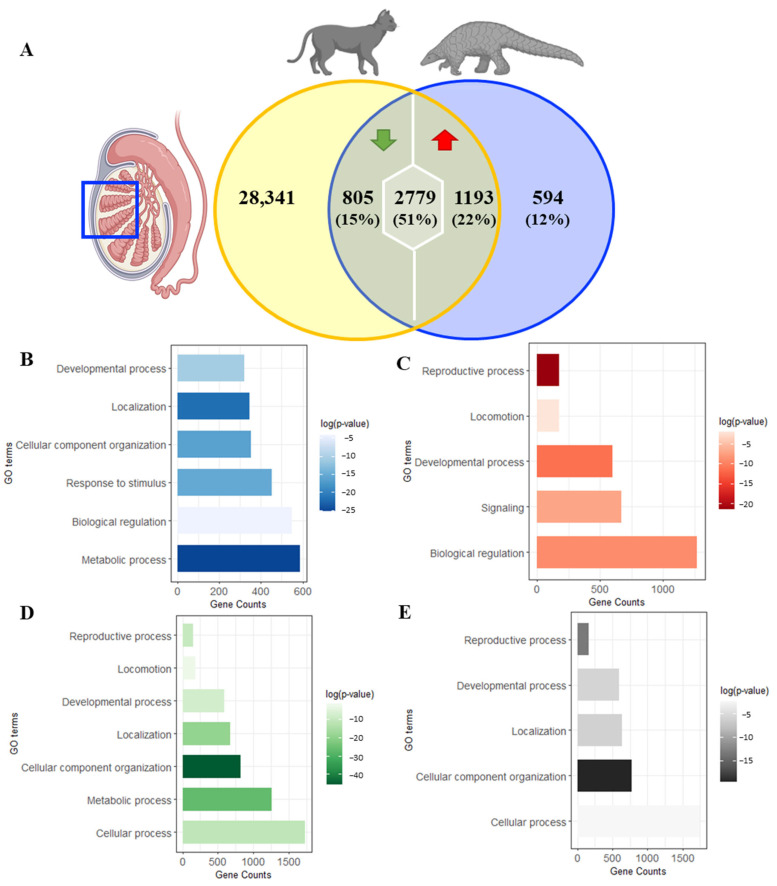
Cross-species gene comparison showed considerable variations between house cats and pangolins. (**A**) Modified Venn diagram of cross-species testicular gene comparisons demonstrated variations in terms of gene expression patterns between the house cats and pangolins; (**B**) pangolin unique genes—Gene Ontology analysis; (**C**) upregulated genes—Gene Ontology analysis; (**D**) downregulated genes—Gene Ontology analysis; (**E**) non-differentially expressed genes—Gene Ontology analysis. Blue box: testis.

As shown in the Venn diagram (Figure 5A), commonly expressed genes (4777 genes) represented 88.9% of the total pangolin testes genes (5371 genes) and only 12% (594 genes) were unique to pangolin testes. Among the commonly expressed genes, 51% (2779 genes) presented a similar expression pattern (non-differentially expressed genes), 22% (1193 genes) were upregulated in pangolin and 15% (805 genes) were downregulated in pangolin when compared with the expression level in house cat. DAVID analysis of general biological processes showed that unique pangolin genes (Figure 5B) were mainly related to metabolic process (gene enrichment *p* value = 1.3 × 10^−11^), upregulated genes (Figure 5C) were mainly related to the reproductive process (gene enrichment *p* value = 5.1 × 10^−10^), and downregulated genes (Figure 5D) and non-differentially expressed genes (Figure 5E) were mainly related to cellular component organization (gene enrichment *p* value = 2.1 × 10^−20^ and 3.7 × 10^−9^, respectively). It is worth noting that we also observed genes that non-differentially expressed genes were strongly related to reproductive process (enrichment *p* value = 1.8 × 10^−6^) (Figure 5E).

### 3.6. In-Depth Analyses Suggests That Domestic Cats May Not Serve as an Ideal Universal Research Model Species for Pangolin

When performing an in-depth analysis of the most enrichment biological processes of each group of genes, similar expression pattern genes were strongly related to the initial steps of male meiosis I processes (meiotic sister chromatid cohesion, formin-nucleated actin cable assembly, activation of meiosis, the establishment of meiotic spindle, and polar body extrusion after meiotic divisions) and hormone secretion (luteinizing hormone secretion and endocrine hormone secretion) (Table 1), whereas upregulated genes were strongly related to the later stages of spermatid differentiation processes (acrosomal vesicle exocytosis, acrosome reaction, sperm axoneme assembly, acrosome assembly, and spermatid development) (Table 2). Furthermore, non-differentially expressed genes were strongly expressed in spermatogonium, spermatozoon, and spermatocyte, whereas upregulated genes were strongly expressed in mature sperm, specifically head plasma membrane, tail, and acrosome. On the other hand, pangolin unique genes were strongly related to metabolic processes. The in-depth analysis shown in Table 3 allowed for us to reveal which substances, such as vitamin K, keratan sulfate, and steroid hormone, were highly metabolized in pangolin testes (Table 3), and lipid metabolism (arachidonic acid and triglyceride biosynthetic process and unsaturated fatty acid metabolic process) was also highly active in the pangolin when compared with the house cat.

General Gene Ontology analysis was performed on the non-differentially expressed genes. Genes related to the reproductive process were isolated and used for an in-depth analysis of their biological processes, tissue expression, and subcellular localization.

General Gene Ontology analysis was performed on the upregulated pangolin genes. Genes related to the reproductive process were isolated and used for an in-depth analysis of their biological processes, tissue expression, and subcellular localization.

General Gene Ontology analysis was performed on the pangolin unique genes. Genes related to metabolic processes were isolated and used for an in-depth analysis of their biological processes, tissue expression, and subcellular localization.

## 4. Discussion

Pangolins are the only representant of their order Pholidota. As of 2014, the Chinese pangolin (*Manis pentadactyla)* has become a critically endangered species, and more than 80% of the population is expected to disappear in the next three generations [2]; similarly, reports in 2005 suggested that its subspecies Formosan pangolin (*Manis pentadactyla pentadactyla*) is headed towards a slow but sure extinction pattern [4]. Despite these facts, limited information is available regarding pangolin reproduction. Given the limited number of animals and a minuscule amount of ejaculation [12], an overview of the male reproductive tract and finding a model species would provide valuable knowledge to guide the scientific effort toward a better understanding of pangolin reproduction. The order Carnivora is the closest order to the pangolin, making cats and dogs the potential model species for pangolin research [13]. To address the abovementioned issues, we reported the first description of pangolin testis and epididymis transcriptome as transcriptome profiling of the male reproductive tract may provide valuable insights into the molecular mechanisms underlying spermatogenesis and male reproductive biology [24], and explored the fitness of carnivores (domestic cats) as a model species for future pangolin research.

In the present study, we observed that the pangolin male reproductive tract exhibited a tissue-specific gene expression, as principal component analysis results showed a distinct gene expression pattern between the testis, caput, and cauda. In the cross-tissue comparison, the number of testis-specific genes was six times higher than the other two parts of the epididymis, and not surprisingly, those genes were essential for spermatogenesis (i.e., *RNF32*, *UBE2R2*) [25], Golgi to acrosome formation (i.e., *RNF111*) [26,27], or were responsible for regulating DNA-binding or damaged DNA repair processes (i.e., *HMBOX1*, *ASCC2*) [28,29]. Furthermore, in the pathway analysis, although the three tissues presented common main pathways, testis exhibited unique pathways, such as Hedgehog, TGF-beta, Ubiquitin-mediated proteolysis, and mTOR pathways; these highly active signaling pathways have been shown to be critical for either the development of the testis, the secretory function of Leydig and Sertoli cells, the organization of peritubular myoid cells, and the maintenance of spermatogenesis as well as blood–testis barrier integrity [30,31].

On the other hand, results from RNAseq analysis highlighted the idea of the epididymal transition as cauda presented a considerable amount of region-specific highly expressed genes. Moreover, Gene Ontology analysis showed that a significant number of genes were identified within extracellular vesicles/exosomes and had protein-binding ability. These results suggest that the protein binding process is one of the highly active biological processes upon epididymal transition in the pangolin epididymis, likely via extracellular vesicle transportation, as suggested in other species [31,32,33,34,35,36]. Indeed, in other species, extracellular vesicles of the epididymis (epididymosomes) have proven to be a source of epigenetic modification for sperm cells upon epididymal transition and modulate sperm–oocyte interaction, capacitation, acrosome reaction, and motility [37,38].

With a limited number of pangolins, it is unlikely to perform in-depth research on this endangered species; thus, the use of model species is an imminent need to advance current knowledge regarding pangolin conservation and is preferred to palliate the low availability of animals or their samples [12]. Domestic cats, belonging to the order Carnivora, are one of the closest domestic species to pangolin [13]; however, it is critically important to validate the adequacy and fitness of a species of interest scientifically before further actions are taken. Pangolin ejaculates consist of only a few microliters with relatively low sperm concentration [12], which greatly complicates the possibility of performing proteomic analysis or cryopreservation of their ejaculates. Fortunately, testicular and epididymal tissues from fresh carcasses are often available from road-killed individuals, making the testis, the organ of sperm production, a great representant for model species assessment. The cross-species comparison revealed that 80% of pangolin genes with known function also appeared in cats, and 52% of the pangolin testicular genes showed similar expression patterns as in house cats. However, further analysis showed that non-differentially expressed genes were mainly related to the first stage of spermatogenesis, and pangolin upregulated genes were strongly correlated with later stages of spermatid differentiation processes. These results indicated that genes regulating spermatogenesis at earlier stages might be similar between domestic cats and pangolins; however, once spermiogenesis starts, gene expression of the two species starts to differ, potentially leading to greater differences in mature sperm cells. These results are in line with the fact that mature spermatozoon is one of the most taxonomically diverse and rapidly evolving cell types among species [39].

Furthermore, genes uniquely present in pangolin were strongly related to metabolism, particularly to the metabolism of vitamin K, keratan sulfate, and steroid hormone. Vitamin K deficiency reduces testosterone production in the testis through downregulation of Cyp11a, a cholesterol side chain cleavage enzyme in rats [40], suggesting a potential difference in hormonal regulation, specifically testosterone, between the two species. Notably, lipid metabolism was also highlighted in our analysis, especially arachidonic acid, triglyceride, and unsaturated fatty acid. Lipid composition, especially lysophospholipids, was a critical component in inducing in vitro spermatogenesis in mice [41]; however, the addition of lysophospholipids cannot successfully induce in vitro spermatogenesis in cats [42]; this is likely due to their differences in lipid metabolism nature; thus, we hypothesize that if in vitro spermatogenesis in pangolin were to be tested, adjusting the concentration of those abovementioned lipids may render better chances of success. Overall, our current findings suggested that domestic cats may not be an adequate model species for the study of male pangolin reproduction; however, whether other carnivore species such as dogs (*Canis lupus familiaris*) are suitable as a model species requires further validation.

## 5. Conclusions

The present research lays the groundwork for the study of pangolin male reproduction. We described for the first time the complete transcriptome of the pangolin male reproductive tract, demonstrating a clear tissue-specific gene expression and identifying critical genes at different reproductive organs. Furthermore, the domestic cat was evaluated for its use as a potential model species for pangolin male reproduction, despite demonstrated the unfitness, as clear differences in spermatid differentiation and metabolism were observed; this critical piece of evidence could lead to a better understanding of pangolin male reproduction and may advance pangolin conservation and gamete preservation in the future.

## Figures and Tables

**Table 1:** In-depth analysis of non-differentially expressed genes related to the reproductive process.

Non-Differentially Expressed Genes Domestic Cat–Pangolin (Reproductive Processes)		Strength	*p* Value
Biological processes	Meiotic sister chromatid cohesion	2.12	3.1 × 10^−5^
	Formin-nucleated actin cable assembly	2.12	1.7 × 10^−2^
	Luteinizing hormone secretion	2.12	1.7 × 10^−2^
	Activation of meiosis	1.94	2.4 × 10^−2^
	Establishment of meiotic spindle	1.82	3.3 × 10^−2^
	Polar body extrusion after meiotic divisions	1.82	3.3 × 10^−2^
	Male meiosis I	1.52	1.4 × 10^−4^
	Endocrine hormone secretion	1.52	1.1 × 10^−2^
Tissue expression	Spermatocyte	1.82	4.9 × 10^−3^
	Spermatozoon	1.72	4.5 × 10^−4^
	Spermatogonium	1.69	8.8 × 10^−3^
Subcellular localization	P granule	1.57	2.9 × 10^−3^
	Condensed nuclear chromosome	1.19	4.8 × 10^−3^
	Acrosomal vesicle	1.05	4.8 × 10^−3^

**Table 2:** In-depth analysis of pangolin upregulated genes related to reproductive process.

Upregulated Genes Pangolin (Reproductive Processes)		Strength	*p* Value
Biological processes	Acrosomal vesicle exocytosis	1.81	4.8 × 10^−3^
	Acrosome reaction	1.53	1.8 × 10^−4^
	Sperm axoneme assembly	1.46	3.8 × 10^−5^
	Acrosome assembly	1.41	3.6 × 10^−3^
	Spermatid differentiation	1.38	4.6 × 10^−27^
	Spermatid development	1.38	4.7 × 10^−26^
	Flagellated sperm motility	1.35	9.1 × 10^−15^
	Germ cell development	1.31	6.5 × 10^−31^
Tissue expression	Testis	0.73	2.1 × 10^−7^
	Internal male genital organ	0.66	5.3 × 10^−7^
	Male reproductive system	0.65	2.1 × 10^−7^
Subcellular localization	Sperm head plasma membrane	2.08	1.9 × 10^−3^
	Acrosomal membrane	1.48	2.8 × 10^−2^
	Sperm flagellum	1.15	1.9 × 10^−3^

**Table 3:** In-depth analysis of pangolin unique genes related to metabolic process.

Unique Genes Pangolin (Metabolic Processes)		Strength	*p* Value
Biological processes	Vitamin K metabolic process	1.68	6.4 × 10^−3^
	Keratan sulfate metabolic process	1.55	1.2 × 10^−3^
	Steroid hormone biosynthesis process	1.49	1.5 × 10^−5^
	Prostaglandin biosynthesis process	1.48	1.98 × 10^−3^
	Arachidonic acid metabolic process	1.42	5.9 × 10^−8^
	Triglyceride biosynthetic process	1.38	3.1 × 10^−2^
	Retinol metabolic process	1.37	9.5 × 10^−7^
	Retinoic acid metabolic process	1.3	7.0 × 10^−3^
	Phosphatidylserine metabolic process	1.3	4.7 × 10^−2^
	Unsaturated fatty acid metabolic process	1.29	1.7 × 10^−9^

## Data Availability

The datasets used and/or analyzed during the current study are available from the corresponding author upon reasonable request.

## Supplementary Materials

The following supporting information can be downloaded at: https://www.mdpi.com/article/10.3390/ani14172592/s1, Table S1: Individual sample information. Table S2: Owner’s consensus statement.
