# Supplementary material for: Transcriptomic Characterization of Male Formosan Pangolin (Manis pentadactyla pentadactyla) Reproductive Tract and Evaluation of Domestic Cat (Felis catus) as a Potential Model Species
Source: Animals (Basel). 2024 Sep 6;14(17):2592. doi: 10.3390/ani14172592 (PMC11394312; doi:10.3390/ani14172592)
Supplement: Supplementary file 1 [file animals-14-02592-s001.zip › animals-3091160-supplementary.pdf]

Supplementary Table S1: Individual sample information

Sample ID	Sample collection (year/month/date)	Age*	Breed	Body condition scoring (BCS)
Pangolin 1	21/11/07	Adult	*Manis pentadactyla pentadactyla*	BCS 3
Pangolin 2	21/12/03	Adult	*Manis pentadactyla pentadactyla*	BCS 4
Pangolin 3	22/02/01	Adult	*Manis pentadactyla pentadactyla*	BCS 4
Pangolin 4	22/02/15	Adult	*Manis pentadactyla pentadactyla*	BCS 3
Pangolin 5	22/11/28	Adult	*Manis pentadactyla pentadactyla*	BCS 3
Pangolin 6	23/01/07	Adult	*Manis pentadactyla pentadactyla*	BCS 3
House cat 1	22/09/07	11 months	Mixed	BCS 4
House cat 2	22/08/09	5 months	Munchkin	BCS 4
House cat 3	22/09/14	11 months	Ragdoll	BCS 4

*Adult was defined as body weight >2350 g, body length >50±5 cm, and tail length >33±2 cm

Supplementary Table S2: Owner’s consensus statement

Animal Research Ethics Committee
National Taiwan University Veterinary Hospital
No. 153, Sec 3, Kee-lung Road, Taipei, Taiwan 106, R.O.C
Phone: 2739-6828

Clinical Trial/Research Approval
National Taiwan University Veterinary Hospital

Date of approval：

NTUVH No.：
Title of protocol：
Department/ Principal Investigator：
Date of application ：

Director
